# Effects of Rating Training on Inter-Rater Consistency for Developing a Dental Hygiene Clinical Rater Qualification System

**DOI:** 10.3352/jeehp.2007.4.5

**Published:** 2007-12-20

**Authors:** Jeong Ran Park, Jung Sook Oh, Moungae Chae, Jae Yeon Jung, Sung Suk Bae

**Affiliations:** 1 Department of Dental Hygiene, Masan College, Masan, Korea.; 2 Department of Dental Hygiene Chunnam Techno College, Gokseong, Korea.; 3 Korean Dental Hygienists' Association, Seoul, Korea.; 4 Department of Dental Hygiene, Hanyang Women's College, Seoul, Korea.; 5 Department of Dental Hygiene, Seoul National University Hospital, Seoul, Korea.

**Keywords:** Clinical competence, Evaluation, Criteria, Item, Difficulty, Inter-rater consistency

## Abstract

We tried to develop itemized evaluation criteria and a clinical rater qualification system through rating training of inter-rater consistency for experienced clinical dental hygienists and dental hygiene clinical educators. A total of 15 clinical dental hygienists with 1-year careers participated as clinical examination candidates, while 5 dental hygienists with 3-year educations and clinical careers or longer participated as clinical raters. They all took the clinical examination as examinees. The results were compared, and the consistency of competence was measured. The comparison of clinical competence between candidates and clinical raters showed that the candidate group's mean clinical competence ranged from 2.96 to 3.55 on a 5-point system in a total of 3 instruments (Probe, Explorer, Curet), while the clinical rater group's mean clinical competence ranged from 4.05 to 4.29. There was a higher inter-rater consistency after education of raters in the following 4 items: Probe, Explorer, Curet, and insertion on distal surface. The mean score distribution of clinical raters ranged from 75% to 100%, which was more uniform in the competence to detect an artificial calculus than that of candidates (25% to 100%). According to the above results, there was a necessity in the operating clinical rater qualification system for comprehensive dental hygiene clinicians. Furthermore, in order to execute the clinical rater qualification system, it will be necessary to keep conducting a series of studies on educational content, time, frequency, and educator level.

## INTRODUCTION

In Korea, dental hygienists take charge of tasks such as scaling, fluoride application for preventing dental caries, and other works related to prevention of dental and oral diseases. To this end, they may join radiography works for dental/oral diagnosis in healthcare or medical institutions equipped with radiographic units for diagnosis to comply with Safety Management Standards based on Article 32-2, Section 1, Korean Medical Act [1]. Since first executed in 1971, the Korean Dental Hygienist Licensing Examination has been conducted in two categories-i.e., written examination and clinical skill test as provided in Article 8, Act Enforcement Rule on Medical Technicians. There is a total of 18 theoretical subjects across 5 fields in dental hygienics. From November, 2004, 30 written items on skills (60 points) and one item of clinical skills (scaling, 40 points) were introduced [2]. The Korean Dental Hygienist Licensing Examination has great significance in the sense that it aims to correctly screen qualified persons as dental hygienists from undergraduates majoring in dental hygienics who want to get registered and licensed through the nationally authorized examination [3-8]. However, many reports have pointed out the problems of a clinical skill test, since it was confined to simple scaling technique. Moreover, some reports have pointed out reliability issues, from inter-rater differences in qualifications in evaluation, as well as lack of education and training for evaluation. They found that current clinical skill tests for dental hygienists have some critical problems in playing a role as a useful measurement tool for Korean dental hygienists to perform their own major clinical duties in the field [9]. Thus, this study focused on reviewing current clinical skill tests for qualifying dental hygienists and identifying the issues for correct evaluation of knowledge and technique, so that it can help raters screen dental hygienists. The purposes of this study are: first, to improve the items of the Korean Dental Hygienist Licensing Examination and thereby to develop items and evaluation criteria for measurement of comprehensive individual clinical competence; second, to develop a standardized evaluating system.

In this study, clinical skill test methods were planned with the following four major steps.

## MATERIALS AND METHODS

A total of 5 researchers (including a senior researcher) were asked to join this study. They consisted of 3 incumbent College professors in dental hygiene, 1 representative of the Korean Dental Hygienists Association (KDHA), and 1 dental hygienist working as a clinical staff member for a university dental hospital. Next, a total of 15 candidates were finally selected (1 or 2 persons per campus) from 13 of 27 campuses that joined the Korean Dental Hygienist Licensing Examination in 2003. Here, the final candidates were selected as novice dental hygienists who already had graduated from a dental hygiene course in Feb. 2004 and have built 1-year clinical careers. Moreover, this study intended to go through sharing feedbacks and having consultative meetings with researchers and thereby develop a rater-screening system by considering minimum interrater errors. The number of raters was extended to 5, which is equal to 3 plus the 2 raters required for Korean Dental Hygienist Licensing Examination. Here, they consisted of 3 dental hygienists working in a clinical division (having a 3-year clinical careers or longer) and 2 dental hygienists (as adjunct instructors) who took charge of the clinical dental hygiene course in the dental hygiene program. In this study, a university dental hospital clinic was used as a place for the dental hygiene clinical test. In order to evaluate comprehensive clinical competence, this study used 5 units of mannequins (mounted on each dental unit chair), a full set of periodontal instruments, and 2 units of mannequins with an artificial calculus attached to the tooth surface.

Calculus detection and probing depth measurement are required courses to examine and identify periodontal conditions to efficiently maintain periodontal health. So, this study devised a new item on the clinical skill test, which was not ever suggested before: detecting and recording artificial calculus attached on dentiform by means of the #11/12 Explorer. Compared with existing ratings on the operation of one dental instrument, the number of clinical skill test items increased to a total of 4 items: 3 items refer to rating operation of the dental instrument, and 1 item refers to rating each candidate's competence to detect artificial calculus. Each candidate's clinical competence was evaluated using the mannequin mounted on a dental unit chair. In addition to existing clinical skill test items, this study adopted basic skill items (patient position, operator position, and dental light position) and extra items such as bracket height and use of mouth mirror (for indirect vision). Conventional 3-level scoring (all correct, one incorrect of 2 or 3 items, all incorrect on 2 or 3 items) was further subdivided into itemized evaluating content. This study developed rating content and criteria to evaluate candidates' competence to maneuver dental instruments such as the Probe (for pocket depth measurement) and #11/12 Explorer (for calculus detection). Detecting and recording artificial calculus attached on dentiform by means of the #11/12 Explorer, which is an unprecedented item on a clinical skill test, was additionally adopted as a new clinical skill test item to evaluate candidates' competence to detect calculus.

All 5 raters who were selected to evaluate candidates' clinical competence were asked to join an orientation in evaluating itemized contents developed by researchers herein for each dental instrument (3 instruments: Probe, #11/12 Explorer, and Curet) and were also told about evaluating procedures. In addition, special education was conducted for raters after completing the first and second clinical examinations for candidatesto reduce the error in clinical raters.

The Clinical Dental Hygiene Examination and Rater Qualification Test was done as follows: first, the whole process of the clinical examination consisted of performing basic skill items (by candidates), evaluating basic skill items (by raters), implementing dental instruments (by candidates) and evaluating a candidate's competence to operate dental instruments (by raters-with Probing, #11/12 Explorer and Curet). Second, the second clinical examination for candidates was conducted in the same ways as the above one. Contrary to the first clinical examination, a total of 5 candidates were asked to join the second test, but 5 raters were asked to join the evaluation as in the first test. Third, 2 weeks after the first and second clinical examinations for the candidates were completed, one researcher provided reorientation in evaluating criteria (as classified for each instrument and evaluation item) for a total of 5 raters. The purpose of this reorientation was to find out whether it was possible to increase the inter-rater consistency in the third clinical. Fourth, 1 week after the first orientation for raters was completed, the third clinical examination for candidates was conducted in the same ways as the first and second clinical examinations for candidates. Contrary to the first and second clinical examinations, a total of 6 candidates were asked to join the third clinical examination, and 5 raters participated in evaluating.

## RESULTS

Based on results from classified evaluation criteria on the candidates' comprehensive clinical competence according to instrument and evaluation items, we sought to determine the degree of difficulty for each item and improve the discriminative power for clinical competence. Based on mean scores obtained in basic skill items and instrumentation competence items, which involved candidates and clinical raters, we compared the clinical competence between candidates and clinical raters so that it could provide useful material on the qualification criteria for raters. In addition, in order to reduce the margins of error between raters who evaluated candidates' clinical competence, we conducted analysis based on the first, second, and third evaluations so that it could provide useful material to develop a clinical rater qualification system.

It was found that raters had higher clinical competence in 3 major clinical procedures than candidates-i.e., probing depth, calculus detection, and calculus removal. Overall, the results comparing clinical competence between candidates and raters showed that raters scored about 1 point (20%) higher in these 3 clinical procedures than candidates (Table 1). In rating the Probe, it was found that most candidates had the highest difficulty in both patient positioning and stroke skill. In rating the Explorer, it was found that most of them revealed relatively high difficulty in instrument insertion and stroke. And in rating Curet application, it was found that most of them had difficulty performing instrument insertion and stroke (Table 2).

Comparing the degree of difficulty in evaluation criteria of candidates' clinical competence, it was found that the most difficult item for Probe was that "The end of Probe tip on proximal surface is not slanted fully to Col area." For evaluation of Explorer clinical competence in anterior teeth surfaces, it was found that "The end of Probe tip on proximal surface is not slanted fully to Col area" was the the most difficult item. For evaluation of Curet instrumentation competence, it was found that "The lower 1/3 section of Curet blade on proximal surface does not reach to Col area" was the most difficult item.

In terms of low inter-rater consistency for each instrument type (Probe, Explorer, and Curet), it was found that almost all instrumentation competence criteria showed low inter-rater consistency in rating the Probe. It was also found that the items related to low inter-rater consistency were those of instrumentation competence rather than basic skill items. In particular, it was found that there were higher inter-rater errors in evaluation criteria on insertion and stroke than any other items. Based on these results, it will be necessary to set the priority and significance of instrument insertion or stroke higher in rater orientation than they are now.

After the first, second, and third basic skills and instrumentation competence tests were completed, we compared itemized inter-rater consistency (Table 3). The itemized evaluation criteria were found to be statistically significant. First, in terms of instrumentation competence in using the Curet on the mesial surface, repeated evaluation and rater reorientation had significant effects on the Stroke item (P<0.001). In terms of instrumentation competence in using the Explorer, repeated evaluation or rater reorientation had no significant effect on the Stroke item (P<0.001). In order to reduce inter-rater errors and improve inter-rater consistency, it will be necessary to develop more objective and formulated rating standards on evaluation items, and also to prepare long-term educational courses on Stroke items or make several rater reorientation courses available on a short-term basis. For using the Probe on the distal surface, patient positioning (as a basic skill) and insertion (under instrumentation competence in using the Curet) showed significant inter-rater consistency (P<0.005). In addition, for patient positioning with the Probe in use, second test showed higher inter-rater consistency than the first evaluation. And in the case of using the Curet, the first evaluation showed higher inter-rater consistency than the second. These findings indicate that evaluation of patient positioning with the Probe was affected by the number of repeated evaluations based on same evaluation criteria, but the number of repeated evaluations with insertion of the Curet didn't have any significant effect on inter-rater errors. However, both evaluation items showed higher inter-rater consistency in the third test after rater reorientation than in the first evaluation before reorientation. They were all influenced by rater reorientation. For insertion, as one item of instrumentation competence using the Curet on the mesial surface. Both the number of repeated evaluations and rater reorientation had no significant effect on improving inter-rater consistency.

According to a comparison of artificial tartar detection skills between candidates and raters, it was found that the mean score of Group A (7 of 15 candidates), Group B (8 of 15 candidates), and the Rater group (consisting of 5 raters) reached 50%, 65%, and 80%, respectively. On the other hand, it was found that the regions with the highest difficulty index in detecting artificial calculus were represented by the upper left molar distolingual surface, lower right molar mesiobuccal surface, and upper right molar distolingual surface. Also, the result of comparison of calculus detection skills between the candidate and rater groups showed that the rater group scored higher than the candidate group (Table 4).

## DISCUSSION

In order to evaluate the comprehensive clinical competence of candidates, this study applied basic skills in addition to instrumentation competence, which were organized into existing evaluation items. It also established clinical skill test settings with head-fixed mannequins mounted on dental unit chairs, because simulating the settings of a patient's oral cavity to the utmost and periodontal treatments via effective appliance operation without causing damage to periodontal tissues is required. However, incorrect application causes discomfort to patients and the wrong operational stance, which may lead to dental hygienists' own occupational disease. In addition, we evaluated candidates' clinical competence of 3 major dental instruments as well as their ability to detect artificial calculus. We adopted additional instruments for evaluation, because 3 major dental instruments were typically employed to carry out the comprehensive dental hygiene process. Clinical competence was classified broadly into two categories, basic skill and instrumentation competence, and corresponding itemized evaluation criteria. It was noted that Stroke, as one item of instrumentation competence, was the evaluation item that showed the identically highest difficulty degree in all three major instruments. The Stroke works by insertion of a fine dental instrument into periodontal pockets requires very subtle and proficient instrumentation competence. Items such as "reaching out instrument up to Col area", "applying stronger lateral pressure", "applying Stroke with instrument handle rotated around line angle area", and "insertion with the inside of Curet blade's lower 1/3 not facing toward tooth surface" were difficult ones. It is expected that these results will be useful as reference to determine evaluation scores depending on itemized difficulty degree and the difficulty degree of evaluation criteria. There was a necessity in the operating clinical rater qualification system for comprehensive dental hygiene clinicians. Furthermore, it will be necessary to provide sufficient practices and education in dental hygiene curriculum so that candidates can improve clinical competence for those evaluation items.

## Figures and Tables

**Table 1:**
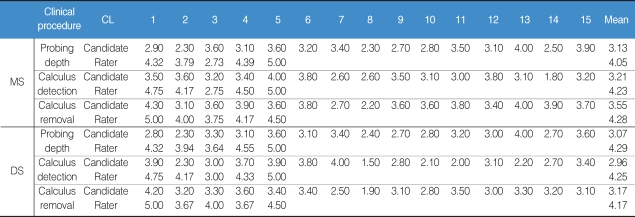
Comparison of clinical competence between candidates and clinical raters

CL: Classification, MS: Mesial Surface, DS: Distal Surface.

**Table 2:**
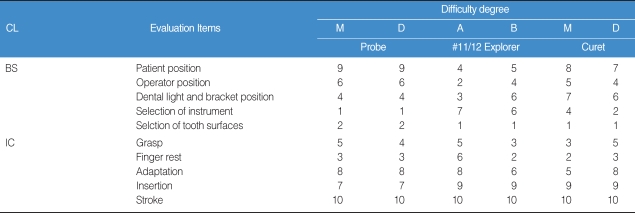
The order of difficulty on candidates' instrumentation competence

CL: Classification, BS: Basic Skills, IC: Instrumentation Competence, M: Mesial, D: Distal, A: surface toward the operator, B: surface away from the operator. The higher the number, the more the difficulty.

**Table 3:**
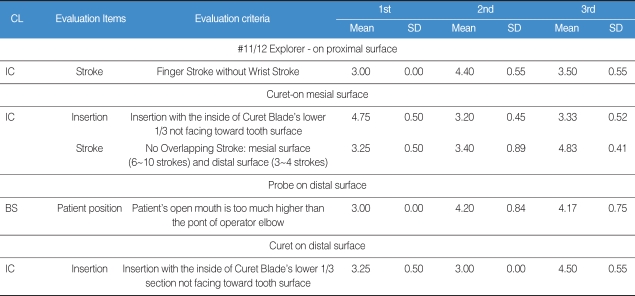
Comparison on inter-rater consistency in the 1st, 2nd and 3rd evaluation

CL: Classification, BS: Basic Skills, IC: Instrumentation Competence.

**Table 4:**
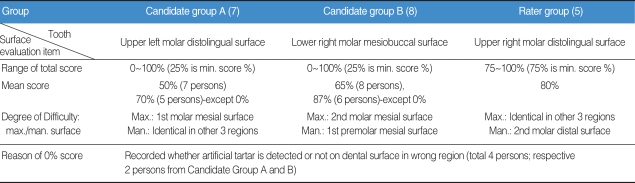
Comparison in calculus detection competence between candidates and raters
